# Evaluation of the Clinical Effects and Frequency of *MEFV* Gene Mutation in Patients with Inflammatory Bowel Disease

**DOI:** 10.1155/2021/5538150

**Published:** 2021-11-15

**Authors:** S. Sahin, D. Gulec, S. Günay, C. Cekic

**Affiliations:** ^1^Clinical Biochemistry, Çiğli State Hospital, İzmir, Turkey; ^2^Tissue Typing Laboratories, Health Sciences University, Tepecik Training and Research Hospital, İzmir, Turkey; ^3^Department of Gastroenterology, Katip Çelebi University, Atatürk Training and Research Hospital, 35360 İzmir, Turkey; ^4^Department of Gastroenterology, Tınaztepe University, School of Medicine, İzmir, Turkey

## Abstract

**Background:**

The clinical and pathological features of inflammatory bowel disease (IBD) and Familial Mediterranean Fever (FMF) are similar.

**Objective:**

Here, the frequency of Mediterranean Fever (MEFV) gene mutation and its effect on the outcome of IBD were evaluated.

**Methods:**

DNA sequence analysis detected the variants on the MEFV gene in patients with IBD. The relationship between mutations and the need for steroids, immunomodulators, biologics, and surgery was assessed.

**Results:**

We evaluated 100 patients with IBD (55 with ulcerative colitis (UC) and 45 with Crohn's disease (CD)) and 60 healthy individuals as controls. The frequency of *MEFV* gene mutation was 26.7% (*n* = 12) and 14.5% (*n* = 8) for UC and CD, respectively. No relationship was found between *MEFV* gene mutation and the need for steroids, immunomodulators, and biologics (*p* = 0.446; *p* = 0.708; *p* > 0.999, resp.); however, in UC, the need for surgery in those with mutation (*p* = 0.018) and E148Q mutation alone was significant (*p* = 0.037).

**Conclusion:**

The rate of *MEFV* gene mutations was high in patients with UC who required surgery. These patients have frequent and severe attacks, indicating that the mutations are related to disease severity. *MEFV* mutation as a modifier factor of IBD should be considered.

## 1. Introduction

The pathogenesis of inflammatory bowel diseases (IBD), including ulcerative colitis (UC) and Crohn's disease (CD), is affected by environmental factors, leading to an uncontrolled immune response in genetically sensitive people [1]. Several studies have investigated the association of diseases with genes, providing a better understanding of their immunopathogenesis. The *NOD2/CARD15* (nucleotide oligomerization domain 2/caspase recruitment domain 15) gene is responsible for the synthesis of proteins that activate the nuclear factor kappa B (NF*κ*B), which has a role in apoptosis and innate immune response, and the susceptibility gene mutations of *NOD2/CARD15* are the first to be associated with IBD [2]. More than 30 IBD-prone genes have been identified, even if their role is less well known [3]. A meta-analysis examining three major genome-wide association studies (GWAS) related to CD reported that mutations with a deep-rooted relationship with CD constitute only 20% of the genetic variations in CD, meaning there is another genetic infrastructure to be explored [4].

Mediterranean Fever (*MEFV*) gene mutations are associated with Familial Mediterranean Fever (FMF) disease, which is an autosomal recessive disorder characterized by fever, serosal inflammation, and recurrent episodes [5]. The *MEFV* and NOD2/CARD15 genes are localized to the same chromosome 16p13. The product of *MEFV* gene, pyrin protein, and NOD2/CARD15 gene product protein are similar in structure. They belong to the same protein family (death domain superfamily), include a common CARD domain, and play a role in the regulation of apoptosis, cytokine release, and inflammation [6].

Thus, it is important to remember that the inflammation load may be increased in patients with IBD who are carriers of the *MEFV* mutation. It is noteworthy that mutations may contribute to secondary amyloidosis, and with early diagnosis, colchicine prophylaxis may be beneficial in such cases.

Because *MEFV* gene mutations play a role in controlling inflammation, in this study, we determine the frequency of mutations in IBD and investigate the effects of mutation presence on the course of the disease.

## 2. Materials and Methods

### 2.1. Patients' Selection

In the study, we enrolled 100 IBD patients (55 with UC and 45 CD) who were followed up by the IBD Unit, Department of Gastroenterology, İzmir Katip Çelebi University, and 60 healthy controls (HC).

### 2.2. Exclusion Criteria

We excluded patients with less than one-year follow-up and those with FMF.

### 2.3. Study Design

We recorded the following data for all the patients: age of onset; disease duration; disease localization; requirements for steroid, immunomodulator, and biologic usage; the need for surgical intervention; and extraintestinal manifestations. Montreal classification and European Crohn's and Colitis Organization (ECCO) guidelines were employed to determine disease localization and disease types, such as UC and CD [7]. In terms of remission, the disease severity, the immunomodulator and biologic usage, and the need for surgery were evaluated according to ECCO guidelines (patients showing disease activity at least two times a year were considered to have frequent recurrent diseases) [8].

### 2.4. Laboratory Analysis

Blood samples (2 cc from each patient) were collected in EDTA-containing tubes using the QIAamp DNA Blood Mini Kit (Qiagen, Germany). DNA was isolated from peripheral blood leukocytes using a standard procedure. Polymerase chain reaction (PCR) was performed to amplify the targeted gene using Applied Biosystems 9700 Thermal Cycler. Oligonucleotide synthesis was conducted to amplify the second and tenth exons of the *MEFV* gene.

Automated DNA sequencing reaction was performed using the ABI PRISM BigDye Terminator v3.1 Cycle Sequencing Kit (Applied Biosystems, USA) according to the manufacturer's instructions. Purified PCR samples were run for 45 min using POP-7 polymer by ABI 3130xl Genetic Analyzers automated capillary electrophoresis device. DNA sequences were analyzed using the SeqScape v2.6 software.

### 2.5. Statistical Analysis

The Statistical Package for the Social Sciences, version 22.0, was utilized in the analyses. Continuous variables were expressed as means, standard deviations, medians, and min–max values, whereas categorical variables were expressed as frequencies and percentages. Chi-squared and Fisher's exact tests were conducted to compare two categorical variables; however, the differences between two independent samples' means or medians were compared using the Mann–Whitney *U* test or median test. Logistic regression analysis was employed to assess the relationships between two or more variables. *p* < 0.05 was considered statistically significant.

### 2.6. Ethical Considerations

The İzmir Bozyaka Training and Research Hospital Institutional Ethics Committee approved the study protocol. All patients were informed about the contents of the study, and their written and verbal informed consent was obtained.

## 3. Results

### 3.1. Patient Characteristics

In this study, we evaluated 100 patients with IBD (55 with UC and 45 CD) and 60 HC. The mean age of patients with IBD and HC was 44.0 ± 14.0 and 41.2 ± 13.2 years, respectively. The IBD group had 50 females (50%), whereas the control group had 30 females (50%). No statistically significant differences were observed between the IBD and control groups in terms of age and gender (*p* > 0.05). The demographic and clinical characteristics of the patients and control groups are shown in Table 1.

### 3.2. Mutation Variants and Frequencies

The ratios of FMF-related mutations (M694V, E148Q, V726A, M680I(G-C), M694I, A744S, R761H, and K695R) in the UC, CD, and HC groups were 14.5%, 26.7%, and 15%, respectively. No statistically significant differences were detected in terms of mutation frequency between the UC and CD groups and the control group (*p* > 0.05). For the patients and HC, FMF-related mutation frequency detected in *MEFV* gene exons 2 and 10 and homozygous and heterozygous changes and frequency of mutation types are demonstrated in Tables 2(a) and 2(b), respectively. The most common mutation in CD is M694V (6.6%), while in UC, it is E148Q (3.6%). Upon evaluating all patients for allele frequency, the most common mutations were M694V (3.5%) and E148Q (3%) (Table 3).

### 3.3. The Effects of Mutations on Clinical Parameters

To examine the effect of *MEFV* gene mutations on phenotypic and clinical variables such as disease behavior and disease activity, patient groups were divided into mutation+ and nonmutation subgroups. Upon assessing the demographic and clinical characteristics of mutation+IBD patients, the number of patients with UC who require surgical intervention was found to be statistically significant compared to those without mutations (*p* = 0.018).

Among the different treatment groups (steroids, biological agents, etc.), none of the medical treatments were associated with the presence of mutations. Moreover, the mutations had no significant effect on the other clinical and phenotypic parameters (Table 4). Using logistic regression analysis, the relationship between mutation types and the need for surgical intervention was evaluated with the following results. The association of E148Q (*p* = 0.036) and M694V (*p* = 0.036) mutations with patients requiring surgery was found to be statistically significant. The presence of the E148Q and M694V mutations increased the need for surgery 6.25 times (OR: 6.25; 95% CI: 1.1–34.6). On the other hand, in patients with UC, the presence of the E148Q mutation alone was significantly associated with the need for surgery (*p* = 0.037); however, for patients with CD, no significant association was detected. Furthermore, in all groups, there was no relationship between the other demographic and phenotypic characteristics and mutation types.

## 4. Discussion

Recently, attempts have been made to classify autoinflammatory diseases based on molecular pathophysiology, and the concept of systemic autoinflammatory diseases has been introduced [9]. Many patients with IBD may develop systemic extraintestinal symptoms, not limited to the gastrointestinal tract. Extraintestinal manifestations can affect almost any organ system, potentially harming the quality of life and functional status of the patients.

First, we investigated the effects of *MEFV* gene mutation on the clinical course and severity of IBD. No significant relationship between extraintestinal symptoms and mutations was found. Previous research suggested that the presence of mutation increases the probability of extraintestinal involvement [10]; however, other studies reported that there were indications of this relationship [11, 12]. In terms of the effect of *MEFV* gene mutations on the clinical course, in addition to demographic data, we analyzed the clinical and prognostic parameters such as disease pattern and need for surgical intervention and potent treatments. Upon reviewing the existing literature, it was observed that in CD, the severity of the disease increased with the presence of *MEFV* mutations; moreover, in some case reports, clinical remission could not be achieved with conventional treatments other than biological agents [13]. In some IBD cases, which carry the *MEFV* mutation, the disease activity could not be controlled until the colchicine treatment was initiated [14, 15]. However, in this study, only in the UC group, surgical requirement rates were higher in patients with *MEFV* gene mutations than those without mutation. M694V and E148Q mutations were more frequently found in patients who require surgery. In contrast, in the CD group, there was no indication that mutations increase the need for surgery. This can be explained by the fact that the CD group was predominantly composed of patients with inflammatory behavior that does not require surgery, and most of these patients do not have mutations.

Second, we evaluated the frequency of *MEFV* gene mutations in IBD using a candidate gene approach, identifying genes and variants that increase susceptibility to the disease. In this study, we focus on the frequency of *MEFV* gene mutations in IBD, rather than the cooccurrence or frequency of FMF in IBD. Only two studies discovered that *MEFV* gene mutation was highly frequent in IBD and reported other confusing factors such as kinship marriages or low mutation rates in control groups [16, 17]. However, similar studies demonstrated that the frequency of *MEFV* gene mutations in IBD patients is not significant compared to the healthy population [10–12, 18]. In this study, *MEFV* gene mutation frequency in patient groups (UC: 14.5%; CD:26%) was not statistically different compared to that in the control group (15%).

Most routine examinations investigating mutations in the *MEFV* gene, such as reverse hybridization strip test, allow only studying the common 10–12 mutations. In this study, we scanned 220 variables found in exons 2 and 10, known to be hot spots, using DNA sequence analysis. There are a limited number of studies investigating the relationship between the *MEFV* gene and IBD using DNA sequence analysis [19]. We believe that using DNA sequence analysis strengthens our work. On the other hand, this study had some limitations as follows. We did not evaluate the inflammatory mediators, such as C-reactive protein and fecal calprotectin, in addition to the clinical parameters, when investigating the effect of the presence of the *MEFV* gene mutation on the severity of IBD. At the same time, we did not conduct a correlation analysis to explore the relationship between the presence of mutations and the extent and severity of mucosal lesions which could positively affect the strength of the study. Moreover, the candidate gene approach could be supported by evaluating *MEFV* gene expression in tissue biopsies in IBD. Besides, the lack of long-term data regarding the effect of mutations on the course of the disease and not screening for proteinuria or signs for amyloidosis appear to be a shortcoming of the present study.

As a result, in this study, the frequency rate of *MEFV* gene mutations in IBD was not high compared to the healthy population. Moreover, the presence of *MEFV* gene mutation was found to be greatly associated with patients with UC who require surgery. Considering that these patients have frequent and severe attacks, it should be confirmed whether the mutations are related to clinical severity. A serious complication of IBD and FMF is also secondary amyloidosis, and its frequency has been correlated with *MEFV* mutations in certain studies [20, 21].

In light of this information, the presence of *MEFV* mutation as a modifier factor in IBD should be considered and evaluated in terms of its association with clinical severity and disease complications, such as secondary amyloidosis, especially in patients who do not respond to potent immunosuppressive treatments and require surgery.

## Figures and Tables

**Table 1:** Demographic and clinical characteristics of patients.

		CD (*n* = 45)	UC (*n* = 55)
Demographic characteristics	Age (mean ± std.)	43.6 ± 14.0	45.2 ± 14
	Duration of the disease (year, median)	4 (8)	5 (7)
	Age of onset of the disease (mean ± std.)	37.5 ± 13.9	38.4 ± 14.6

UC localization, *n* (%)	Proctitis	—	2 (3.6)
	Left colitis	—	18 (32.7)
	Extensive colitis	—	35 (63.6)

CD localization, *n* (%)	Ileal	15 (33.4)	—
	Colonic	1 (2.2)	—
	Ileocolic	29 (64.4)	—

CD behavior pattern, *n* (%)	Inflammatory	23 (51.1)	—
	Fibrotic	8 (17.8)	—
	Fistulizing	14 (31.1)	—
	Perianal disease	12 (26.7)	—

Disease activity, *n* (%)	Remission	41 (91.1)	46 (83.6)
	Active	4 (8.9)	9 (16.4)
	Recurrent disease	26 (57.7)	19 (34.5)
	Steroid requirement	34 (75.5)	30 (54.5)
	Immunomodulator use requirement	37 (82.2)	26 (47.2)
	The necessity of using biological agents	20 (44.4)	10 (18.1)
	Surgical requirement	14 (31)	5 (11)

Extraintestinal manifestations, *n* (%)	Arthritis	7 (15.6)	4 (7.2)
	Pyoderma gangrenosum	0	1 (1.8)
	Thromboembolic events	0	1 (1.8)
	Ankylosing spondylitis	2 (4.4)	1 (1.8)
	Absent	36 (80)	48 (87.4)

CD: Crohn's disease; UC: ulcerative colitis; IBD: inflammatory bowel disease.

**Table:** (a) FMF-related mutation frequency in the *MEFV* gene

Mutation	Controls (*n* = 60)	CD (*n* = 45)	UC (*n* = 55)
Absent (*n*, %)	51 (85)	33 (73.3)	47 (85.5)
Present (*n*, %)	9 (15)	12 (26.7)	8 (14.5)
*p* values	NA	0.359	>0.999

CD: Crohn's disease; UC: ulcerative colitis; FMF: Familial Mediterranean Fever; *MEFV*: Mediterranean Fever gene.

**Table:** (b) Frequency of mutation types

Mutation type	UC, *n* (%)	CD, *n* (%)	Controls, *n* (%)
Heterozygous for one	8 (100)	8 (66.8)	9
E148Q/w	4 (50)	2 (17)	3
M694V/w	1 (12.5)	3 (25)	3
R761H/w	0	1 (8.3)	0
V726A/w	1 (12.5)	0	0
M680I/w	0	1 (8.3)	3
K695R/w	1 (12.5)	1 (8.3)	0
R628K/w	1 (12.5)	0	0
Homozygous for one	0	2 (16.6)	0
M694V/M694V	0	1 (8.3)	0
M694I/M694I	0	1 (8.3)	0
Compound heterozygous	0	2 (16.6)	0
M694V/V726A	0	1 (8.3)	0
M680I/V726A	0	1 (8.3)	0

W: wild type; CD: Crohn's disease; UC: ulcerative colitis.

**Table 3:** Allele frequency of *MEFV* mutations in patient and control groups.

*MEFV* mutations	CD alleles, *n* = 90 (%)	UC alleles, *n* = 110 (%)	Control alleles, *n* = 120 (%)
E148Q	2 (2.2%)	4 (3.6%)	3 (2.5%)
M694V	6 (6.6%)	1 (0.9%)	3 (2.5%)
R761H	1 (1.1%)	0	0
V726A	2 (2.2%)	1 (0.9%)	0
M680I	2 (2.2%)	0	3 (2.5%)
K695R	1 (1.1%)	1 (0.9%)	0
R628K	0	1 (0.9%)	0
M694I	0	0	0

CD: Crohn's disease; UC: ulcerative colitis; IBD: inflammatory bowel disease; *MEFV*: Mediterranean Fever gene.

**Table 4:** Effect of *MEFV* mutations on clinical variables.

	Variables		Mutation (+)/*n*	Mutation (-)/*n*	*p* value
UC, *n* = 55	Surgical requirement		3/8	2/47	0.018
	Biologic agent requirement		1/8	9/47	>0.999
	Immunomodulator requirement		3/8	23/47	0.708
	Steroid usage		3/8	27/47	o.446
	Recurrent disease		3/8	16/47	>0.999
	UC localization	Extensive colitis	5/8	30/47	>0.999
		Left colitis (+proctitis)	3/8	17/47	
	Extraintestinal manifestations		7/8	41/47	>0.999
	Age of onset of the disease (mean ± std)		36.0 ± 18.0/8	37.8 ± 16.0/47	0.558

CD, *n* = 45	Surgical requirement		5/12	9/33	0.470
	Biologic agent requirement		6/12	14/33	0.651
	Immunomodulator requirement		11/12	26/33	0.419
	Steroid usage		10/12	24/33	0.699
	Recurrent disease		9/12	17/33	0.158
	CD localization	İleal	5/12	10/33	0.496
		Colonic and ileocolonic	7/12	23/33	
	CD behavior	Fibrotic	3/12	5/33	0.661
		Others	9/12	28/33	
		Fistulizing	3/12	11/33	0.725
		Others	9/12	22/33	
	Perianal disease		3/12	9/33	>0.999
	Extraintestinal manifestations		9/12	27/33	0.682
	Age of onset of the disease (mean ± std.)		33.3 ± 11.8/12	39.1 ± 14.5/8	0.253

CD: Crohn's disease; UC: ulcerative colitis; *MEFV*: Mediterranean Fever gene.

## Data Availability

The data used to support the findings of this study are available from the corresponding author upon request.

## Abbreviations

CARD: Caspase activation and recruitment domains
CD: Crohn's disease
UC: Ulcerative colitis
IBD: Inflammatory bowel disease
ECCO: European Crohn's and Colitis Organization
FMF: Familial Mediterranean Fever
GWAS: Genome-wide association studies
*MEFV*: Mediterranean Fever gene
NOD2/CARD1: Nucleotide oligomerization domain 2/caspase recruitment domain 15
NF*κ*B: Nuclear factor kappa B
PCR: Polymerase chain reaction.
